# Adding Estimates of Central Venous Pressure Boosts the Performance of Non-Invasive Assessment of the Portosystemic Gradient Prior to TIPS Implantation

**DOI:** 10.3390/diagnostics16071091

**Published:** 2026-04-04

**Authors:** Fabian Stoehr, Maximilian Moos, Lukas Müller, Tilla Loew, Annika Merzweiler, Christian Labenz, Tobias Jorg, Simon Johannes Gairing, Peter R. Galle, Roman Kloeckner, Jens Mittler, Michael B. Pitton, Tobias Bäuerle, Felix Hahn

**Affiliations:** 1 Department of Diagnostic and Interventional Radiology, University Medical Center Mainz, 55131 Mainz, Germany; 2 Department of Internal Medicine I, University Medical Center Mainz, 55131 Mainz, Germany; 3 Institute of Interventional Radiology, University Hospital of Schleswig-Holstein—Campus Lübeck, 23538 Lübeck, Germany; 4 Department of General, Visceral and Transplant Surgery, University Medical Center Mainz, 55131 Mainz, Germany

**Keywords:** transjugular intrahepatic portosystemic shunt (TIPS), CT-based scoring systems, portosystemic gradient (PSG), central venous pressure (CVP)

## Abstract

**Background**: Non-invasive scoring systems for predicting the hepatic venous pressure gradient (HVPG) and, thus, clinically significant portal hypertension (CSPH) have been proposed; the aim of this study was to evaluate the accuracy of these scores in a cohort of patients undergoing transjugular intrahepatic portosystemic shunt (TIPS) placement and to further analyze patients without a markedly elevated portosystemic gradient (PSG) at the time of the procedure. **Methods**: We retrospectively analyzed 314 patients who underwent TIPS implantation at our tertiary care center between 2010 and 2022. The diagnostic performance of CT-based scoring systems by Iranmanesh (Score 1) and Kihira (Score 2), as well as laboratory-based scores including MELD (Score 3), FIB-4 (Score 4), and APRI (Score 5), was assessed for detecting a markedly elevated PSG (PSG > 10 mmHg). Additionally, we evaluated whether incorporating the inferior vena cava (IVC) diameter as a surrogate marker of central venous pressure (CVP) improves the accuracy of CT-based scores. **Results**: Both Scores 1 and 2 showed high sensitivity (89–87%) but low specificity (33–27%). ROC analysis revealed AUC values between 0.65 and 0.62. Laboratory-based scores (Score 3–5) performed poorly with AUCs of 0.57–0.54. Adding IVC diameter as an estimator for CVP to Scores 1 and 2 significantly increased the AUC to 0.74 and 0.76. In Lasso regression, IVC diameter was selected as a significant variable for PSG estimation. **Conclusions**: CT-based scoring systems showed promise in assessing markedly elevated PSG, but their specificity was low. Including the IVC diameter improved accuracy in detecting elevated PSG in TIPS patients. Future scoring systems should incorporate CVP estimators like the IVC diameter.

## 1. Introduction

Due to progressive changes in the splanchnic circulation, patients with liver cirrhosis develop an increase in portal pressure during the course of the disease [1]. This increase leads to an elevation of the pressure gradient between the portal vein and the inferior vena cava above the normal range of 1–5 mmHg [2]. When this portosystemic gradient (PSG) rises above 10 mmHg, the number of complications due to portal hypertension, like variceal bleeding and refractory ascites, distinctly increases [2,3]. Those complications include severe events like upper gastrointestinal bleeding resulting from ruptured gastroesophageal varices, which lead to significantly increased mortality among these patients [2]. Furthermore, refractory ascites impairs the quality of life of the patients and can also lead to life-threatening complications like spontaneous bacterial peritonitis or acute kidney injury [4]. Thus, a PSG greater than or equal to 10 mmHg is defined as clinically significant portal hypertension (CSPH) [5,6].

To measure the PSG, invasive access to the portal vein is necessary. During a transjugular intrahepatic portosystemic stent shunt (TIPS) procedure, the portal pressure can be measured directly prior to shunt creation and can be compared to the central venous pressure (CVP), which is recorded via the sheath placed in the right atrium. To avoid invasive procedures, non-invasive methods to identify patients with elevated PSG have been proposed. Several laboratory-based scoring systems, such as the aspartate aminotransferase-to-platelet ratio index (APRI) and the Fibrosis-4 Index (FIB-4), were originally developed for the assessment of liver fibrosis but have also been shown to correlate with the hepatic venous pressure gradient (HVPG) and may therefore be useful for the non-invasive prediction of clinically significant portal hypertension (CSPH) [7,8,9,10,11]. In different validation studies, however, the sensitivity and specificity of the different scoring systems varied significantly [7,8,9,12]. Additionally, none of the scoring systems based on image-derived features include estimates for CVP. However, as the PSG is calculated as the difference between portal pressure and central venous pressure (CVP), we hypothesized that incorporating a surrogate marker of CVP, such as the inferior vena cava (IVC) diameter, could improve non-invasive estimation of the PSG.

Thus, one aim of this study was to evaluate the accuracy of those non-invasive scoring systems in order to predict PSG elevation in a cohort of TIPS patients, for whom directly assessed portal and central venous pressure measurements were available, and to further investigate patients without markedly increased PSG at the time of the TIPS procedure. Secondly, this study aimed to investigate whether incorporation of an estimate for CVP could improve the performance of non-invasive scoring systems to predict elevated PSGs.

## 2. Methods

This retrospective study was carried out according to the Declaration of Helsinki and was approved (permit number 2021-15984) by the local ethics committee (Medical Association of Rhineland Palatinate, Mainz, Germany). Informed consent was waived due to the retrospective nature of the study.

### 2.1. Patients

**Figure 1:**
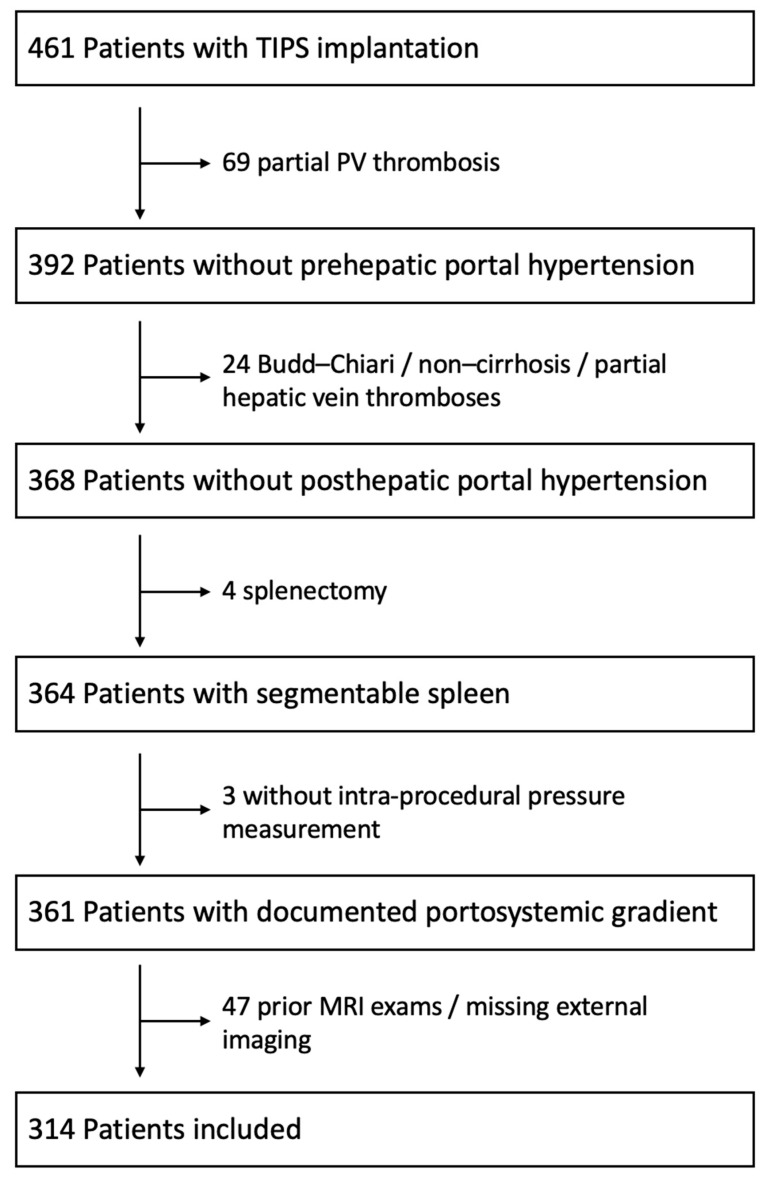
Flow chart for patient selection.

Between January 2010 and December 2022, 461 patients were indicated for TIPS implantation and treated at our tertiary care center. A total of 314 patients fulfilled the inclusion criteria and were identified from our clinical database. The following inclusion criteria were applied: (1) age above 18 years; (2) available CT imaging prior to TIPS treatment; (3) available demographical, clinical, and laboratory data at initiation of the TIPS treatment, including PSG measurement after access to the portal venous system; (4) no identifiable pre- or posthepatic cause for portal hypertension; (5) no splenectomy; (6) no prior TIPS placement; and (7) no liver transplantation. Figure 1 depicts the patient selection and dropouts.

### 2.2. Treatment and Follow-Up

All TIPS procedures were performed according to previously published institutional standards by experienced interventional radiologists and under general anesthesia [13]. Pressure measurements were performed using a 5F-pigtail catheter positioned in the main portal vein, whereas the tip of the 10F-sheath remained in the right atrium. The mean portal vein pressure (PVP) and mean right atrial pressure (RAP) were registered, and the PSG was calculated as the difference between them [13]. Measurements were simultaneously obtained with continuous double line registration as previously reported [13]. Prior to the TIPS procedure all patients underwent extensive clinical examinations, laboratory values were gathered, and the patients underwent contrast-enhanced computed tomography (CT) for treatment planning.

### 2.3. Data Acquisition

All patient data was retrospectively acquired using the local clinical, laboratory, and radiology information system (Mesalvo, Freiburg, Germany) as well as the local picture archiving and communication system (Sectra, Linköping, Sweden). Liver volume from the CT image datasets was assessed using a commercially available solution (syngo.CT Liver Analysis, Siemens Healthcare, Erlangen, Germany). Splenic volume was assessed automatically using an in-house-designed software solution [14,15]. Segmentations were manually reviewed and corrected by two radiologists with long-standing experience in liver imaging (LM and FH) [16]. The final dataset included all available data on patient demographics, clinical assessments of the underlying liver disease, procedural information including the PSG values, and imaging factors and laboratory parameters obtained prior to the TIPS treatment.

### 2.4. Scoring Systems for CSPH Estimation

For the head-to-head comparison of different non-invasive scoring systems based on laboratory and image-derived parameters, five commonly used scoring systems were included. The scores using CT data were that by Iranmanesh (Score 1) and the PH score by Kihira (Score 2) [8,9].

The scores using laboratory data were the Model for end-stage liver disease (MELD) (Score 3), Fibrosis-4 Index (Score 4) and AST-to-platelet ratio index (Score 5), which were calculated as described in their original publications [17,18,19].

The definitions and calculations of the scores are summarized in Table 1.

In a second step, the inferior vena cava (IVC) diameter as an established non-invasive estimate of the CVP, which was measured in CT imaging as previously described, was added to the image-derived Scores 1 and 2 [20]. In a third step, a LASSO regression was used to fit a linear model selecting parameters of Scores 1–5 and the IVC diameter.

### 2.5. Statistical Analysis

All statistical analyses and visualizations were conducted using RStudio (RStudio Team, 2020; RStudio: Integrated Development for R, RStudio, PBC, Boston, MA, USA, http://www.rstudio.com, last accessed on 10 August 2025) with R version 4.0.3 (R Foundation for Statistical Computing, http://www.R-project.org, last accessed on 10 June 2025). Continuous variables are summarized as medians and ranges, whereas categorical and binary variables are presented as absolute counts and percentages. Group comparisons were performed using Fisher’s exact test, the chi-squared test, or the Mann–Whitney U test, as appropriate. The discriminative performance of the different non-invasive methods for detecting elevated PSG was evaluated using the area under the receiver operating characteristic curve (AUC), reported together with the corresponding standard deviation. Comparisons between AUCs were carried out using the DeLong method. Additionally, the continuous correlation between the PSG and the respective scores was assessed using Spearman’s rank correlation coefficient. LASSO regression was performed using the R package “glmnet” with a binomial logistic link function (https://CRAN.R-project.org/package=MatchIt, https://CRAN.R-project.org/package=glmnet, last accessed on 10 June 2025). A *p*-value < 0.05 was considered statistically significant.

## 3. Results

### 3.1. Baseline Characteristics

Of the 314 patients analyzed in this study, 246 (86.5%) were male. The median age at the time of TIPS treatment was 58 years. The mean PSG prior to TIPS was 16 ± 4.8 mmHg. Out of the 314 patients, 45 were indicated for TIPS due to signs of portal hypertension (e.g., ascites, collateral circulation), but at the time of the TIPS procedure, their PSG was ≤10 mmHg.

If patients with a PSG of 9–10 mmHg are considered borderline cases, 19 patients remained with a PSG of ≤8 mmHg. Of these, nine had known concomitant heart failure and/or signs of right heart strain. In four patients, after access to the portal venous system and pressure measurements were obtained, the PSG was so low that a TIPS was not created following ad hoc interdisciplinary discussions.

**Figure 2:**
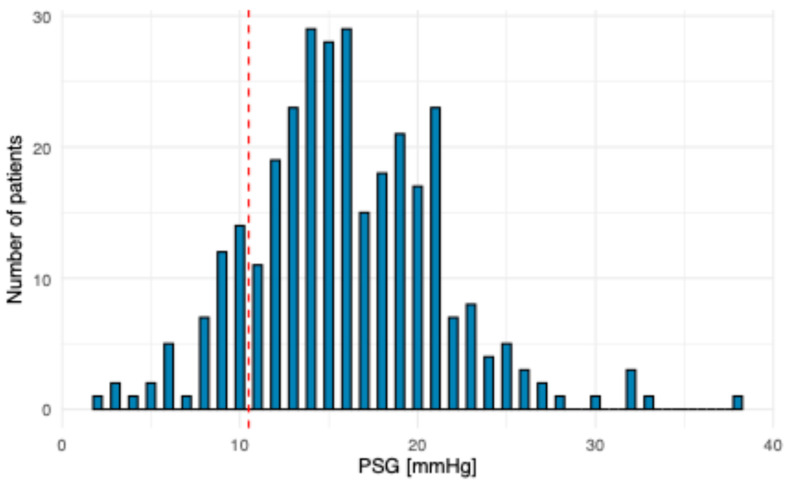
Distribution of PSG measurements among the study population.

Figure 2 displays the distribution of PSG values; Table 2 displays the baseline characteristics of the cohort.

### 3.2. Diagnostic Performance of the Scoring Systems

**Figure 3:**
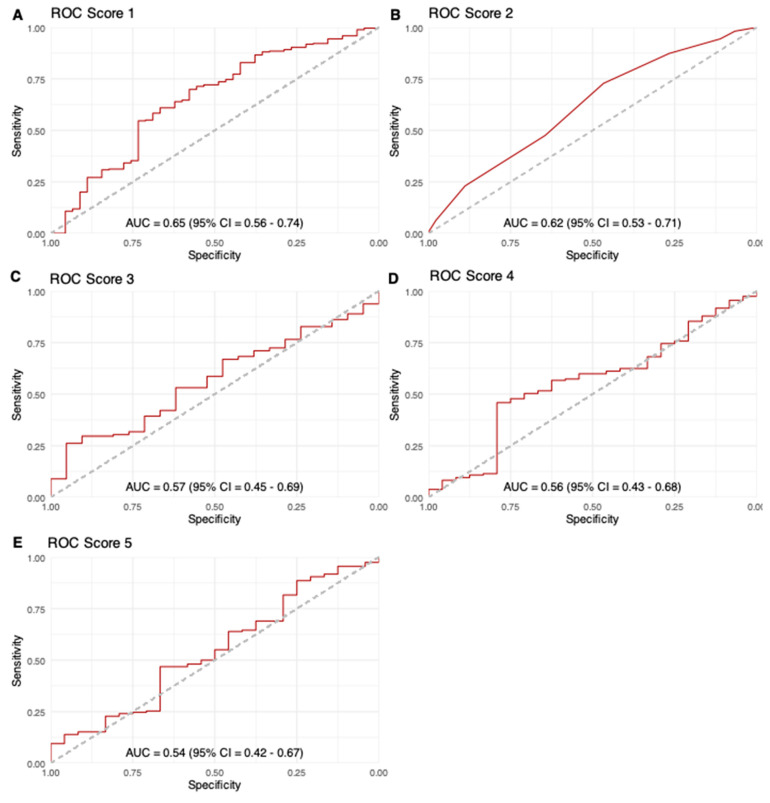
ROC curves for the different scoring systems: (**A**). Score 1, Iranmanesh et al. score; (**B**). Score 2, PH score; (**C**). Score 3, Model for end-stage liver disease (MELD); (**D**). Score 4, Fibrosis-4 Index; (**E**). Score 5, AST-to-platelet-ratio index; AUC, area under the receiver operating characteristic curve.

Regarding the non-invasive identification of patients with elevated PSG, both Score 1 and Score 2 with cut-offs as proposed in the original publications showed good sensitivity (89% and 87%, respectively) but poor specificity (33% and 27%, respectively). ROC analysis yielded AUCs of 0.65 for Score 1 and 0.62 for Score 2; the laboratory scores performed poorly with AUCs of 0.57 for Score 3, 0.56 of Score 4 and 0.54 for Score 5 (Figure 3).

When the DeLong test was used for a head-to-head comparison of the optimized AUCs of the different scoring systems, none of the comparisons reached significance (Supplementary Table S1).

### 3.3. Influence of Adding the IVC Diameter on the Diagnostic Performance of the Image-Derived Scoring Systems

**Figure 4:**
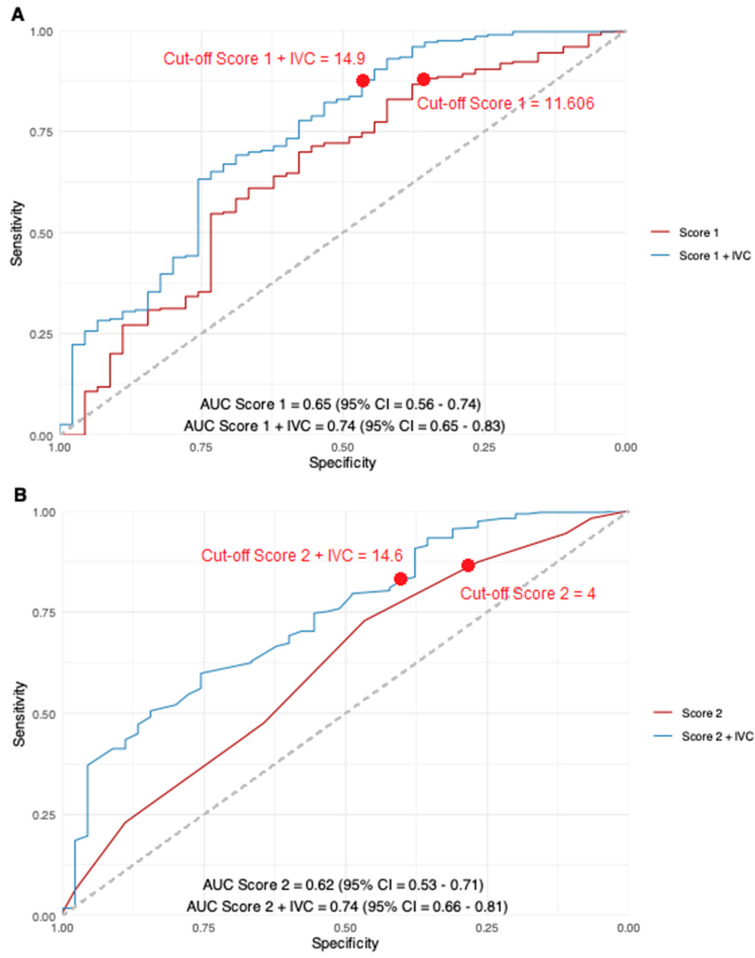
(**A**) Comparison of the ROC curves for Score 1 (Iranmanesh et al.) before and after adding the IVC diameter. (**B**) Comparison of the ROC curves for Score 2 (PH Score) before and after adding the IVC diameter.

In the subgroup of patients with low PSG, central venous pressure (CVP) was significantly increased compared to that for patients with high PSG (13 ± 6.6 mmHg vs. 7.7 ± 4 mmHg, *p* < 0.01). Thus, in a second step, the IVC diameter as an estimator of CVP was added to Score 1 and Score 2 in linear regression. The addition of the IVC diameter resulted in a significant AUC increase to 0.74 and 0.76, respectively (*p* = 0.03 and *p* < 0.01) (Figure 4). The regression formulas and standardized beta coefficients are listed in the supplement (Supplementary Table S2).

Additionally, sensitivity remained high at appropriate cut-off points, while specificity was improved: in the new model Score 1 + diameter of the IVC, the sensitivity was 87% (original Score 1: 89%) and the specificity was 47% (original Score 1: 33%), while in the model Score 2 + diameter of the IVC, the sensitivity was 83% (original Score 2: 87%) and the specificity was 40% (original Score 2: 27%). The corresponding contingency tables are depicted in Table 3.

Spearman’s rank correlation analysis demonstrated overall weak to moderate correlations between the evaluated scores and the pre-TIPS PSG (Supplementary Figure S1).

### 3.4. Lasso Model

**Figure 5:**
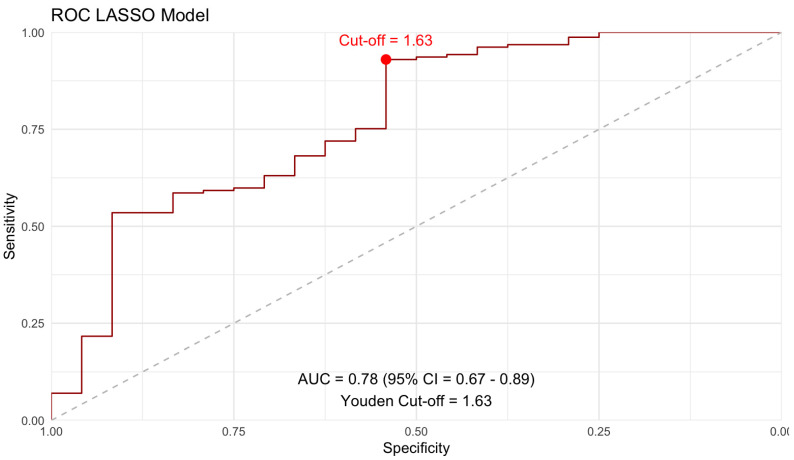
ROC curve of the new LASSO model.

In a third step, LASSO regression was performed on the cohort for estimation of markedly elevated PSG using the parameters of Scores 1–5 and the IVC diameter. A 10-fold cross-validation approach yielded a minimum lambda of 0.015. Subsequently, ROC analysis of the LASSO model was performed, as illustrated in Figure 5.

Of importance, the IVC diameter was retained as a significant parameter in variable selection. The regression formula is provided in the supplement (Supplementary List S1).

## 4. Discussion

This study aimed to compare the performance of non-invasive scoring systems to identify patients with elevated PSG in a cohort of patients prior to TIPS implantation. In summary, scoring systems based on image-derived features outperformed scoring systems relying solely on laboratory values. However, while sensitivity was high for the two imaging-based scores, their specificity remained low. Thus, such scores can only be one component of clinical decision-making.

As a novel aspect, we aimed to improve the performance of the scores by including an indicator for the CVP, which is missing in the existing scoring systems. Adding the IVC diameter as an indicator of CVP to the imaging-based scoring systems yielded significantly increased AUC values. The importance of an estimator for the CVP was corroborated in LASSO regression as well, in which the IVC diameter was selected as a significant parameter contributing to high sensitivity and increased specificity values.

In a first part of our study, we focused on non-invasive scoring systems based on CT image-derived features to identify patients with elevated PSG. The scoring system by Iranmanesh et al. is based on liver and spleen volume and the presence of perihepatic ascites [8]. While the latter can be easily assessed purely visually, volumetry requires manual segmentation of individual CT slices at the time of score generation. In previous works, it was therefore argued that scoring systems based on organ volume would require extensive post-processing and would therefore play a minor role in routine clinical practice [12]. However, with advances in the field of artificial intelligence, automated organ segmentation from CT datasets is possible [14,21,22,23,24]; once this is implemented, organ volumes could be fed easily into the Iranmanesh et al. formula for patients with questionable CSPH. While the initial study reported an AUC of the score of 0.91 in the training cohort and an AUC of 0.82 in the validation cohort, the score performed worse in other validation studies, with an AUC of 0.77 [8,9,12]. In our study, its performance was below these values with an AUC of 0.65. Compared with the original study and the studies mentioned above, a possible explanation for the difference might be the fact that in our study the patients with low PSG values that were misclassified—and thus resulted in the low specificity—did have a TIPS indication clinically and a similar distribution of portal pressure values but had increased CVP values.

Second, we looked at the PH score from Kihira et al. [9]. This score follows an easily reproducible system that consists of the visual qualitative assessment of several surrogate parameters for portal hypertension. These include varices and ascites and the craniocaudal splenic diameter, which has been established as a two-dimensional diameter to estimate the real splenic volume, especially in patients with advanced splenomegaly [25]. In the original publication, the AUCs for the assessment of CSPH were 0.81 and 0.83 for the two observers. Our study showed an AUC of 0.62 with high sensitivity values and poor specificity values. There was no significant difference in the performance of the scores by Iranmanesh et al. and Kihira et al. Looking at the parameters included in the PH score, it is noticeable that the craniocaudal diameter could be assessed automatically or substituted with automated spleen volumetry. In addition, an automated estimation of varices and ascites could be added in the future. Such automation might further increase the performance of the score and enable it to be objectified while at the same time further reducing the effort required [26].

In addition to the parameters of the aforementioned scoring systems, several studies have investigated more advanced measurement techniques for non-invasive estimation of CSPH in various modalities in recent years [27,28]. One method that has shown promising results is quantitative assessment of the liver surface nodularity (LSN) [12]. However, the outcome of this method differs between CT and MRI measurements in the same patient, with a particularly high failure rate in MRI [29,30]. Qualitative standards for the methods have been defined recently; however, clinical validation of these is still pending [31]. The same applies to techniques like elastography or advanced MRI techniques (e.g., 4D flow and relaxometry) [27,28]. All these methods require expertise and additional software solutions. Thus, although highly promising, clinical validation and implementation are still pending. More recently, the NICER model has been introduced as a composite non-invasive approach combining spleen stiffness, liver stiffness, platelet count, and BMI to estimate the probability of clinically significant portal hypertension [32].

Compared to those for the image-based scoring systems, the AUCs for the laboratory-based scoring systems were significantly lower. Moreover, they were lower in our cohort of severely ill patients than the values described in previous validation studies [7,12]. However, it should be mentioned that the values varied greatly between the different validation studies: while values for the AUC of the FIB-4 and APRI scores of 0.77 and 0.79 were described by Sartoris et al., the AUC values in the validation study by Cho et al. were 0.65 and 0.64 and, thus, closer to the values in our study. Overall, it appears that while the scores have important clinical value in assessing liver parenchymal status, they should function more as adjuncts to imaging for the assessment of portal hypertension.

To the best of our knowledge, this is the first study evaluating the potential influence of including a surrogate for CVP on the diagnostic performance of the described scoring systems. We hypothesized that this surrogate might have a positive impact, especially on specificity of image-based scores, as a PSG results from portal pressure and CVP. However, the scoring systems put their focus on the portal signs of an increased PSG. In our cohort, there was no significant difference in portal pressure between patients with and without elevated PSG, but a significant difference between CVP values was observed. By including a surrogate for CVP, namely the largest diameter of the IVC immediately below the right atrium, we were able to improve the AUCs of the imaging scores and demonstrated that IVC diameter was retained as a significant diagnostic factor for PSG estimation in our cohort. Nevertheless, it remains unclear whether other imaging parameters are better suited to assess CVP, and our findings should be investigated in further studies. Moreover, CVP depends on many factors and may additionally be influenced by the anesthetic procedure in the TIPS setting [33]. Recently, however, CVP as a prognostic factor for patients with liver disease has been gaining importance [34]. This should be considered in future work.

Besides its retrospective design, this single-center study has several limitations. First, the cohort consisted of patients with an indication for TIPS and evident signs of portal hypertension. Thus, the investigated cohort differed from the cohorts and the screening nature of the scores’ original publications, which constitutes a spectrum bias. The number of patients with PSG values < 10 mmHg was low, resulting in imbalanced group sizes. In our study, 14 patients had a borderline PSG of exactly 10 mmHg. According to Baveno VII consensus guidelines, CSPH is defined as a PSG ≥ 10 mmHg [5]. However, in our analysis, we adopted the threshold used by Iranmanesh et al., the score relying on the most sophisticated organ segmentations, which defined elevated PSG as >10 mmHg [8]. Consequently, these patients were assigned to the non-elevated PSG group. Except for the four cases with very low PSG, the decision for TIPS implantation in the non-elevated PSG group was made on an interdisciplinary basis, driven by therapy-refractory ascites or variceal bleeding.

In addition, the cohort was predominantly male, and alcoholic etiology was the most prevalent, which may have introduced a source of bias and limited the generalizability and reproducibility of our findings to more diverse patient populations.

Nevertheless, this cohort enabled comparison of scoring systems in a population in which portal venous and central venous pressures were directly measured. Second, pressure measurements were obtained during TIPS placement under general anesthesia, whereas CT imaging was performed without anesthesia, introducing variability in physiological conditions. Third, although CVP was retained as a relevant feature in LASSO regression, external validation of its inclusion in the scoring systems is still pending to confirm its reproducibility.

## 5. Conclusions

The evaluated scoring systems showed promising results in the assessment of portal hypertension; however, due to their limited specificity, they cannot currently replace conventional invasive measurements such as HVPG. In our cohort of TIPS patients, CT-based morphological scoring systems outperformed laboratory-based scores in estimating the PSG. Furthermore, the inclusion of the IVC diameter improved the accuracy, particularly the specificity, of CT morphological scores for detecting elevated PSG. Therefore, estimators of CVP should be incorporated into future scoring models for predicting the PSG, and their potential role warrants further investigation.

## Figures and Tables

**Table 1:** Overview of the evaluated scores.

Name	Definition/Calculation
Iranmanesh	17.37 − 4.91 × ln(liver-to-spleen volume ratio) + 3.8 (if perihepatic ascites is present)
Kihira	Number of variceal sites (0: none; 1: one; 2: two; 3: ≥3 sites) + volume of ascites (0: none; 1: minimal perihepatic/perisplenic fluid; 2: intraperitoneal fluid without significant abdominal wall distension; 3: fluid with significant abdominal wall distension) + maximum craniocaudal spleen diameter (0: <13 cm; 1: 13–15 cm; 2: 15–20 cm; 3: >20 cm)
MELD	9.57 × ln(creatinine [mg/dL]) + 3.78 × ln(total bilirubin [mg/dL]) + 11.2 × ln(INR) + 6.43
Fibrosis-4 Index	$\frac{Age\left[years\right]\times AST\left[\frac{U}{l}\right]}{Plateletcount\left[\frac{10^{9}}{l}\right]\times \sqrt{ALT}\left[\frac{U}{l}\right]}$
AST-to-Platelet Ratio Index	$\frac{AST\left[\frac{U}{l}\right]}{Plateletcount\left[\frac{10^{9}}{l}\right]}$

**Table 2:** Baseline characteristics of the cohort.

Variable	All Patients
Age, years, median (IQR)	58 (51–67)
Sex, n (%)	
Female	68 (13.5)
Male	246 (86.5)
Leading etiology of liver disease, n (%)	
Alcohol	210 (66.9)
HBV	17 (5.4)
HCV	18 (5.7)
MASLD (Metabolic dysfunction-Associated Steatotic Liver Disease)	12 (3.85)
Other	57 (18.2)
Leading indication for TIPS implantation, n (%)	
Ascites	205 (65.3)
Therapy/prophylaxis of variceal bleeding	106 (33.8)
Other	3 (0.9)
Leading indication for TIPS implantation in patients PSG ≤ 10 mmHg, n (%)	
Ascites	31 (68.9)
Bleeding	11 (24.4)
Other	3 (6.7)
Pre-TIPS PSG, mmHg, median (IQR)	16 (13–29)
Post-TIPS PSG, mmHg, median (IQR)	5 (4–7)
Platelets, per nl, median (IQR)	116 (75–170)
Bilirubin, mg/dL, median (IQR)	1.5 (1.0–2.0)
Albumin, g/L, median (IQR)	25 (22–28)
Creatinine, mg/dL, median (IQR)	1.4 (0.9–2.1)
INR, median (IQR)	1.3 (1.2–1.7)
Sodium, mmol/L, median (IQR)	135 (132–139)
MELD score, points, median (IQR)	16 (12–19)
Child Pugh, n (%)	
A	71 (23%)
B	229 (72%)
C	14 (5%)
Average time score-assessment before TIPS, median (IQR)	8 Days (0–13)

**Table 3:** Contingency tables for Score 1 and Score 2 and the extension of those scores using the IVC diameter as a surrogate for the central venous pressure.

Score 1	Elevated PSG	Non-elevated PSG
Elevated PSG predicted	238	30
Non-elevated PSG predicted	31	15
Score 1 + IVC	Elevated PSG	Non-elevated PSG
Elevated PSG predicted	230	24
Non-elevated PSG predicted	36	21
Score 2	Elevated PSG	Non-elevated PSG
Elevated PSG predicted	235	33
Non-elevated PSG predicted	34	12
Score 2 + IVC	Elevated PSG	Non-elevated PSG
Elevated PSG predicted	223	27
Non-elevated PSG predicted	46	18

## Data Availability

The datasets generated and analyzed during the current study are not publicly available due to them containing information that could compromise patient privacy. However, data is available from the corresponding author on reasonable request.

## Supplementary Materials

The following supporting information can be downloaded at https://www.mdpi.com/article/10.3390/diagnostics16071091/s1, Table S1: Head-to-head comparison of the optimized AUCs of the different scoring systems using the DeLong test; Table S2: Regression coefficients for Scores 1 and 2 with the addition of the IVC diameter; List S1: Lasso regression formula; Figure S1. Correlation matrix of Spearman’s rank correlation coefficients between the pre-TIPS PSG, the evaluated scores, and the corresponding models incorporating IVC diameter.

## References

[1] Fernandez M. (2015). Molecular pathophysiology of portal hypertension. Hepatology.

[2] Bosch J., Abraldes J.G., Berzigotti A., García-Pagan J.C. (2009). The clinical use of HVPG measurements in chronic liver disease. Nat. Rev. Gastroenterol. Hepatol..

[3] Tsochatzis E.A., Bosch J., Burroughs A.K. (2014). Liver cirrhosis. Lancet.

[4] Piano S., Tonon M., Angeli P. (2018). Management of ascites and hepatorenal syndrome. Hepatol. Int..

[5] de Franchis R., Bosch J., Garcia-Tsao G., Reiberger T., Ripoll C., Baveno VII Faculty (2022). Baveno VII—Renewing consensus in portal hypertension. J. Hepatol..

[6] de Franchis R. (2015). Expanding consensus in portal hypertension: Report of the Baveno VI Consensus Workshop: Stratifying risk and individualizing care for portal hypertension. J. Hepatol..

[7] Cho E.J., Kim M.Y., Lee J.H., Lee I.Y., Lim Y.L., Choi D.H., Kim Y.J., Yoon J.H., Baik S.K. (2015). Diagnostic and Prognostic Values of Noninvasive Predictors of Portal Hypertension in Patients with Alcoholic Cirrhosis. PLoS ONE.

[8] Iranmanesh P., Vazquez O., Terraz S., Majno P., Spahr L., Poncet A., Morel P., Mentha G., Toso C. (2014). Accurate computed tomography-based portal pressure assessment in patients with hepatocellular carcinoma. J. Hepatol..

[9] Kihira S., Kagen A.C., Vasudevan P., Jajamovich G.H., Schiano T.D., Andrle A.F., Babb J.S., Fischman A., Taouli B. (2016). Non-invasive prediction of portal pressures using CT and MRI in chronic liver disease. Abdom. Radiol..

[10] Duarte-Rojo A., Patel K., Rockey D.C. (2024). Noninvasive assessment of liver fibrosis and portal hypertension. Curr. Opin. Gastroenterol..

[11] Banini B.A., Patel S., Yu J.W., Kang L., Bailey C., Strife B.J., Siddiqui M.S., Patel V., Matherly S.C., Lee H. (2023). Derivation and Validation of a Model to Predict Clinically Significant Portal Hypertension Using Transient Elastography and FIB-4. J. Clin. Gastroenterol..

[12] Sartoris R., Rautou P.E., Elkrief L., Pollorsi G., Durand F., Valla D., Spahr L., Terraz S., Soubrane O., Cauchy F. (2018). Quantification of Liver Surface Nodularity at CT: Utility for Detection of Portal Hypertension. Radiology.

[13] Pitton M.B., Weinmann A., Kloeckner R., Mittler J., Ruckes C., Düber C., Otto G. (2022). Transjugular Portosystemic Stent Shunt: Impact of Right Atrial Pressure on Portal Venous Hemodynamics Within the First Week. Cardiovasc. Intervent. Radiol..

[14] Müller L., Kloeckner R., Mähringer-Kunz A., Stoehr F., Düber C., Arnhold G., Gairing S.J., Foerster F., Weinmann A., Galle P.R. (2022). Fully automated AI-based splenic segmentation for predicting survival and estimating the risk of hepatic decompensation in TACE patients with HCC. Eur. Radiol..

[15] Müller D., Kramer F. (2021). MIScnn: A framework for medical image segmentation with convolutional neural networks and deep learning. BMC Med. Imaging.

[16] Fedorov A., Beichel R., Kalpathy-Cramer J., Finet J., Fillion-Robin J.C., Pujol S., Bauer C., Jennings D., Fennessy F., Sonka M. (2012). 3D Slicer as an image computing platform for the Quantitative Imaging Network. Magn. Reson. Imaging.

[17] Kamath P.S., Kim W.R. (2007). The model for end-stage liver disease (MELD). Hepatology.

[18] Sterling R.K., Lissen E., Clumeck N., Sola R., Correa M.C., Montaner J., Sulkowski M.S., Torriani F.J., Dieterich D.T., Thomas D.L. (2006). Development of a simple noninvasive index to predict significant fibrosis in patients with HIV/HCV coinfection. Hepatology.

[19] Wai C.T., Greenson J.K., Fontana R.J., Kalbfleisch J.D., Marrero J.A., Conjeevaram H.S., Lok A.S. (2003). A simple noninvasive index can predict both significant fibrosis and cirrhosis in patients with chronic hepatitis C. Hepatology.

[20] Griffin M., Ivey-Miranda J., McCallum W., Sarnak M., Eder M., Bellumkonda L., Maulion C., Wilson F.P., Rao V.S., Testani J. (2022). Inferior Vena Cava Diameter Measurement Provides Distinct and Complementary Information to Right Atrial Pressure in Acute Decompensated Heart Failure. J. Card. Fail..

[21] Müller L., Gairing S.J., Kloeckner R., Foerster F., Weinmann A., Mittler J., Stoehr F., Emrich T., Düber C., Galle P.R. (2022). Baseline Splenic Volume Outweighs Immuno-Modulated Size Changes with Regard to Survival Outcome in Patients with Hepatocellular Carcinoma under Immunotherapy. Cancers.

[22] Gul S., Khan M.S., Bibi A., Khandakar A., Ayari M.A., Chowdhury M.E.H. (2022). Deep learning techniques for liver and liver tumor segmentation: A review. Comput. Biol. Med..

[23] Hosny A., Parmar C., Quackenbush J., Schwartz L.H., Aerts H. (2018). Artificial intelligence in radiology. Nat. Rev. Cancer.

[24] Lee C.M., Lee S.S., Choi W.M., Kim K.M., Sung Y.S., Lee S., Lee S.J., Yoon J.S., Suk H.I. (2021). An index based on deep learning-measured spleen volume on CT for the assessment of high-risk varix in B-viral compensated cirrhosis. Eur. Radiol..

[25] Nuffer Z., Marini T., Rupasov A., Kwak S., Bhatt S. (2017). The Best Single Measurement for Assessing Splenomegaly in Patients with Cirrhotic Liver Morphology. Acad. Radiol..

[26] Du Z., Yang L., He H., Wu X., Qi X., Zhang Y. (2025). Artificial intelligence for the noninvasive diagnosis of clinically significant portal hypertension. EngMedicine.

[27] Kennedy P., Bane O., Hectors S.J., Fischman A., Schiano T., Lewis S., Taouli B. (2020). Noninvasive imaging assessment of portal hypertension. Abdom. Radiol..

[28] Wan S., Liu X., Jiang H., Teng Z., Song B. (2021). Noninvasive imaging assessment of portal hypertension: Where are we now and where does the future lie?. Expert Rev. Mol. Diagn..

[29] Lo G.C., Besa C., King M.J., Kang M., Stueck A., Thung S., Wagner M., Smith A.D., Taouli B. (2017). Feasibility and reproducibility of liver surface nodularity quantification for the assessment of liver cirrhosis using CT and MRI. Eur. J. Radiol. Open.

[30] Besa C., Wagner M., Lo G., Gordic S., Chatterji M., Kennedy P., Stueck A., Thung S., Babb J., Smith A. (2018). Detection of liver fibrosis using qualitative and quantitative MR elastography compared to liver surface nodularity measurement, gadoxetic acid uptake, and serum markers. J. Magn. Reson. Imaging.

[31] Sartoris R., Lazareth M., Nivolli A., Dioguardi Burgio M., Vilgrain V., Ronot M. (2020). CT-based liver surface nodularity for the detection of clinically significant portal hypertension: Defining measurement quality criteria. Abdom. Radiol..

[32] Jachs M., Odriozola A., Turon F., Moga L., Téllez L., Fischer P., Saltini D., Kwanten W.J., Grasso M., Llop E. (2024). Spleen stiffness measurement by vibration-controlled transient elastography at 100 Hz for non-invasive predicted diagnosis of clinically significant portal hypertension in patients with compensated advanced chronic liver disease: A modelling study. Lancet Gastroenterol. Hepatol..

[33] Ushinsky A., Kim D., Darcy M., Kim S.K. (2023). Impact of General Anesthesia on the Right Atrial Pressure During Transjugular Intrahepatic Portosystemic Shunt Creation: A Propensity Score Match Analysis. Cardiovasc. Intervent. Radiol..

[34] Bommena S., Mahmud N., Boike J.R., Thornburg B.G., Kolli K.P., Lai J.C., German M., Morelli G., Spengler E., Said A. (2023). The impact of right atrial pressure on outcomes in patients undergoing TIPS, an ALTA group study. Hepatology.

