# Supplementary material for: Adding Estimates of Central Venous Pressure Boosts the Performance of Non-Invasive Assessment of the Portosystemic Gradient Prior to TIPS Implantation
Source: Diagnostics (Basel). 2026 Apr 4;16(7):1091. doi: 10.3390/diagnostics16071091 (PMC13072961; doi:10.3390/diagnostics16071091)
Supplement: Supplementary file 1 [file diagnostics-16-01091-s001.zip › diagnostics-4221299-supplementary.pdf]

Supplementary Table S1. Head-to-head comparison of the optimized AUCs of the different scoring systems using the DeLong test.

Scores	Score 1	Score 2	Score 3	Score 4	Score 5
**Score 1**	X	0.40	0.27	0.22	0.18
**Score 2**	0.40	X	0.50	0.42	0.35
**Score 3**	0.27	0.50	x	0.95	0.85
**Score 4**	0.22	0.42	0.95	X	0.66
**Score 5**	0.18	0.35	0.85	0.66	X

Supplementary Table S2. Regression coefficients for Scores 1 and 2 with the addition of the IVC diameter.

Predictor	Estimate	Standardized	Std. Error	t value	p value	Significance
**Score 1**						
(Intercept)	19.08130	–	1.95905	9.740	< 2e-16	***
Iranmanesh	0.24399	0.16504	0.07998	3.051	0.00248	**
IVC diameter	−0.33479	−0.25050	0.07231	−4.630	5.38e-06	***
**Score 2**						
(Intercept)	18.37724	–	1.79251	10.252	< 2e-16	***
PH_score	0.78653	0.24043	0.17432	4.512	9.12e-06	***
IVC diameter	−0.31482	−0.23556	0.07122	−4.421	1.36e-05	***

Estimate: Unstandardized regression coefficient. Indicates the expected change in the outcome variable for a one-unit change in the predictor, holding all else constant. Standardized: Regression coefficient based on z-transformed predictors, allowing for comparisons of relative effect sizes across variables. Asterisks indicate significance levels: *** $p < 0.001$, ** $p < 0.01$.

Supplementary List S1. Lasso regression formula.

inv. Logit(5.161885 + 0.0006364571 · ALT − 0.001900994 · Platelets + 0.00983411 · Age + 0.00002460887 · Craniocaudal spleen diameter + 0.2213552 · Varices score (according to PH score) + 0.0005631662 · Spleen volume − 0.2121494 · IVC diameter)

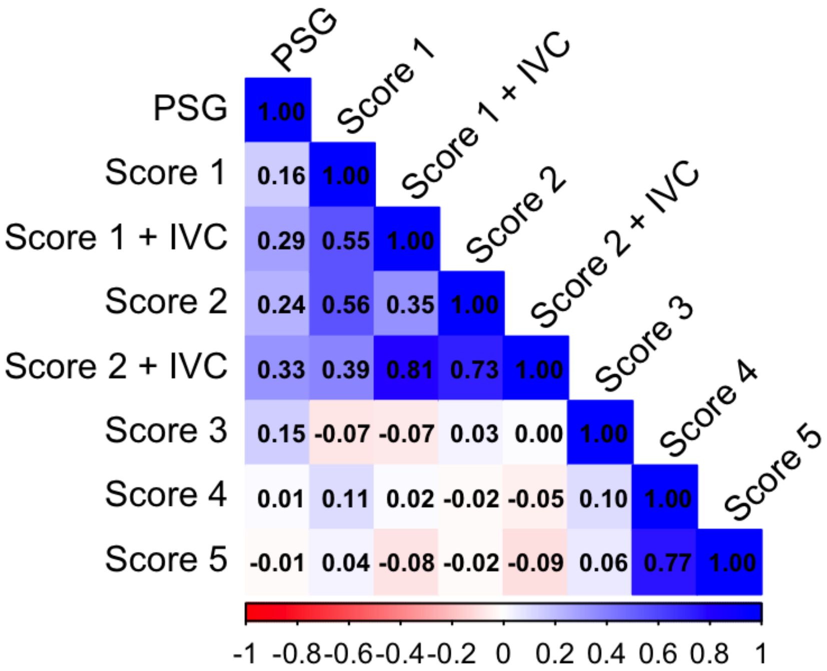

Supplementary Figure S1. Correlation matrix of Spearman's rank correlation coefficients between the pre-TIPS PSG, the evaluated scores, and the corresponding models incorporating IVC diameter.
